# Supplementary figures and images for: Baculoviruses manipulate host lipid metabolism via adipokinetic hormone signaling to induce climbing behavior
Source: PLoS Pathog. 2025 Jan 31;21(1):e1012932. doi: 10.1371/journal.ppat.1012932 (PMC11819524; doi:10.1371/journal.ppat.1012932)

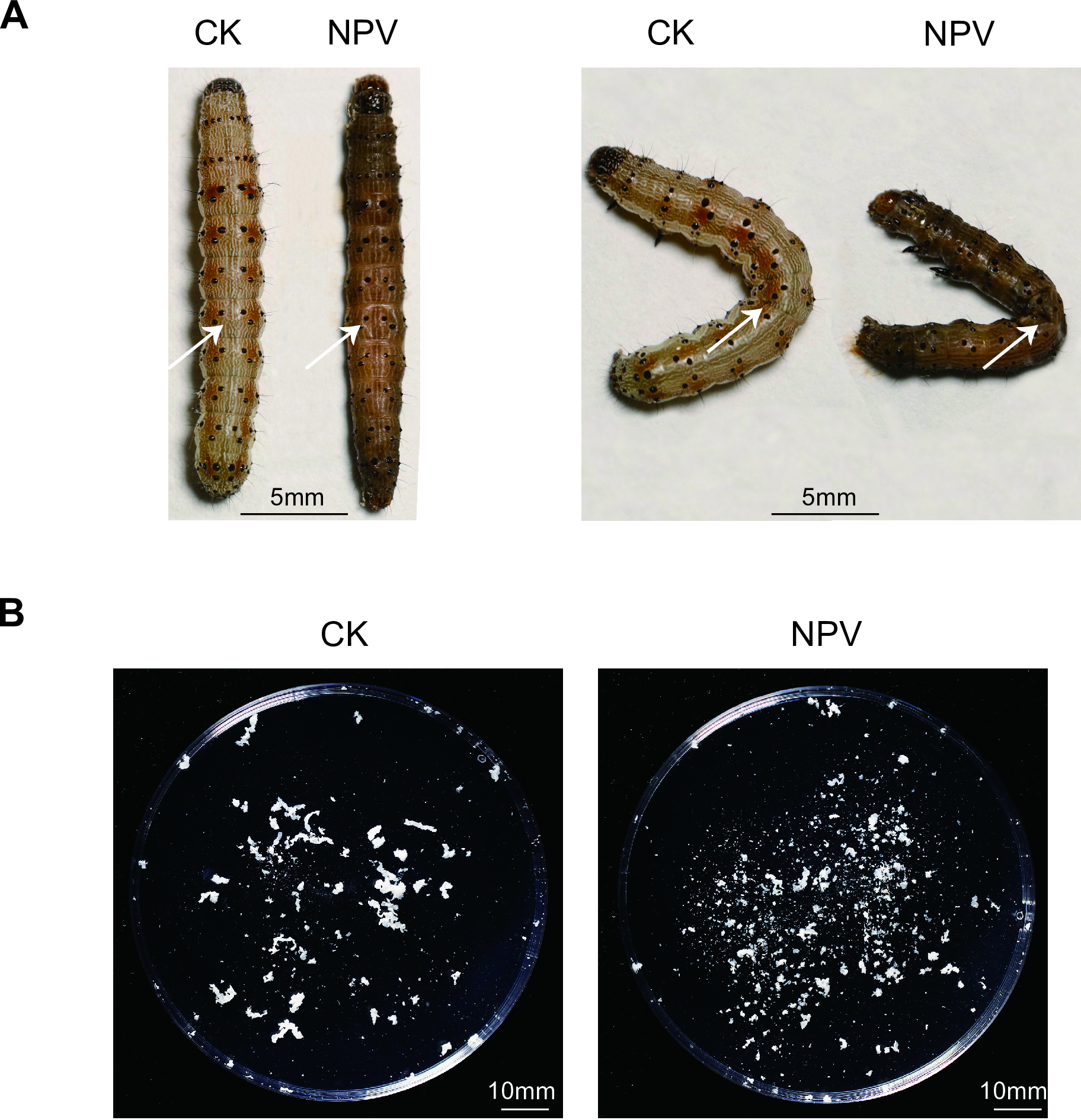

Supplement: S1 Fig — (A) Photos of the healthy (CK) and HearNPV-infected (NPV) 4th instar H. armigera larvae. The arrow indicated that the body of infected larva is softer than the healthy larva. Scale bar: 5 mm. (B) Photos of fat bodies of CK and NPV larvae. Scale bar: 10 mm. (TIF) [file ppat.1012932.s001.tif]

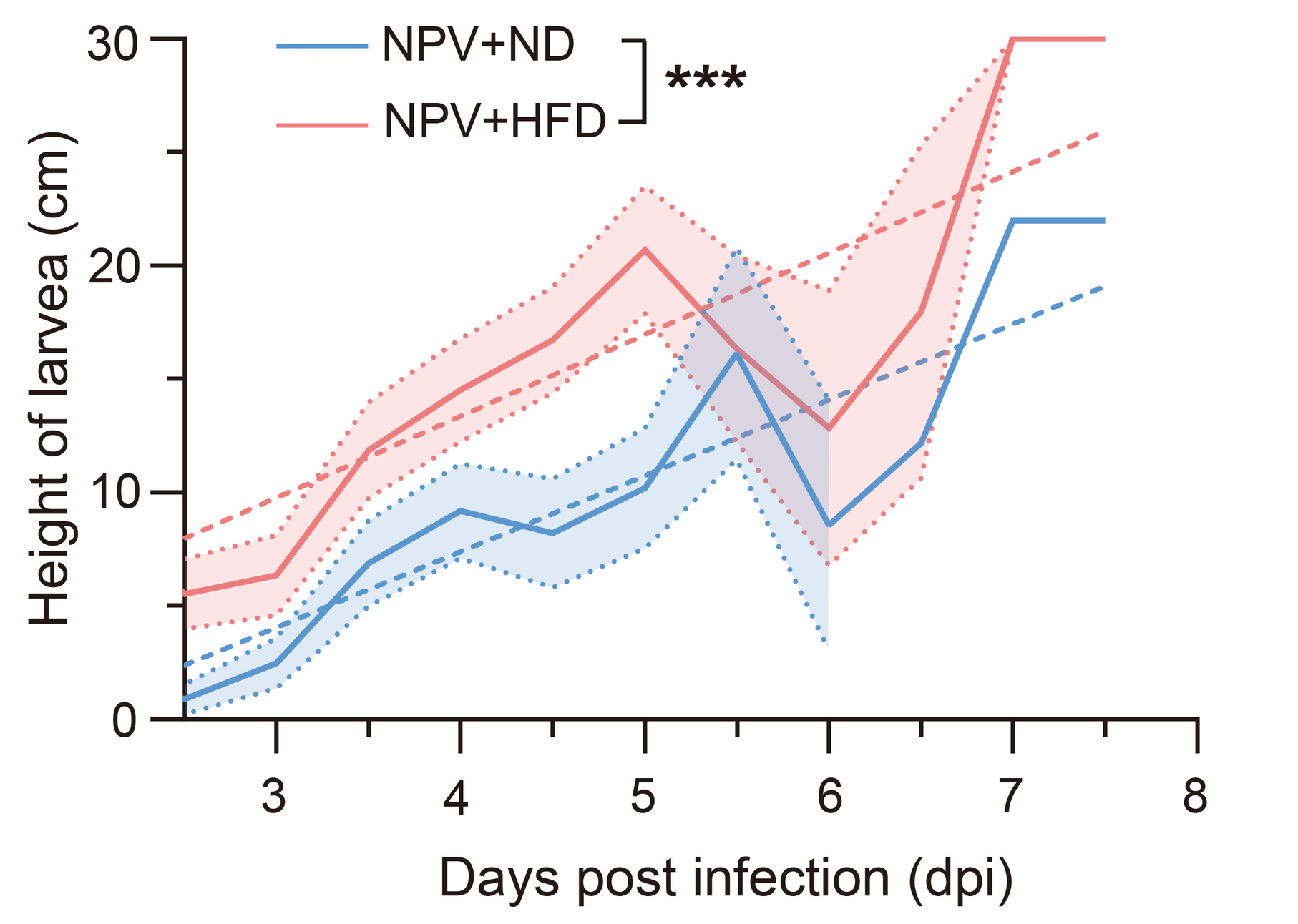

Supplement: S2 Fig — Mean (±SEM) height of infected larvae with normal-diet (NPV+ND) and infected larvae with high-fat-diet (NPV+HFD). ***p < 0.001. (TIF) [file ppat.1012932.s002.tif]

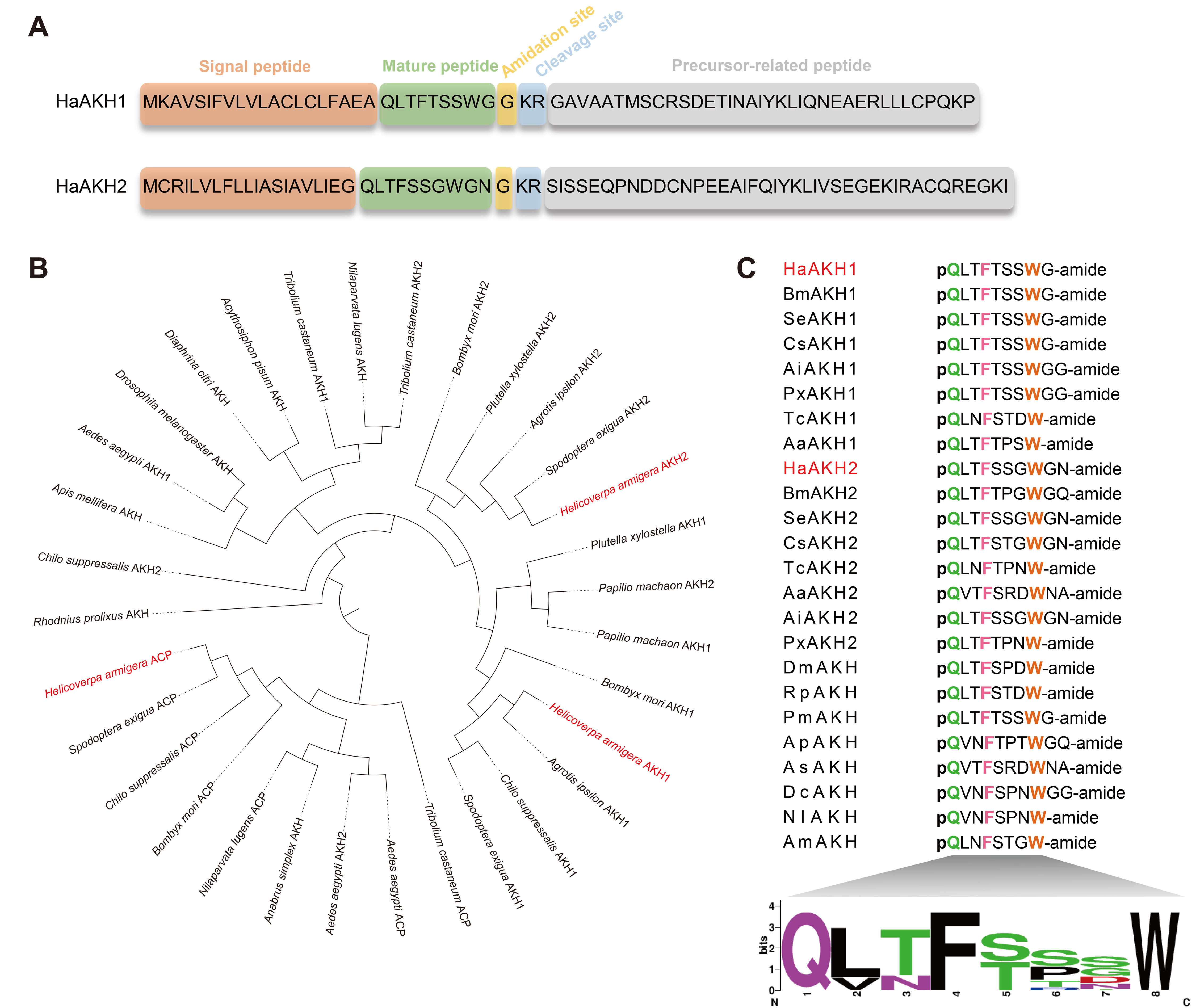

Supplement: S3 Fig — (A) Schematic diagram showing protein characteristics for HaAKH1 and HaAKH2. (B) Phylogenetic analysis of AKHs of insect species. H.armigera sequences were highlighted in red. (C) Alignment of sequence of AKH mature peptides in different species. The calculated consensus logo was shown at the bottom. The protein names and accession numbers were listed in S2 Table. (TIF) [file ppat.1012932.s003.tif]

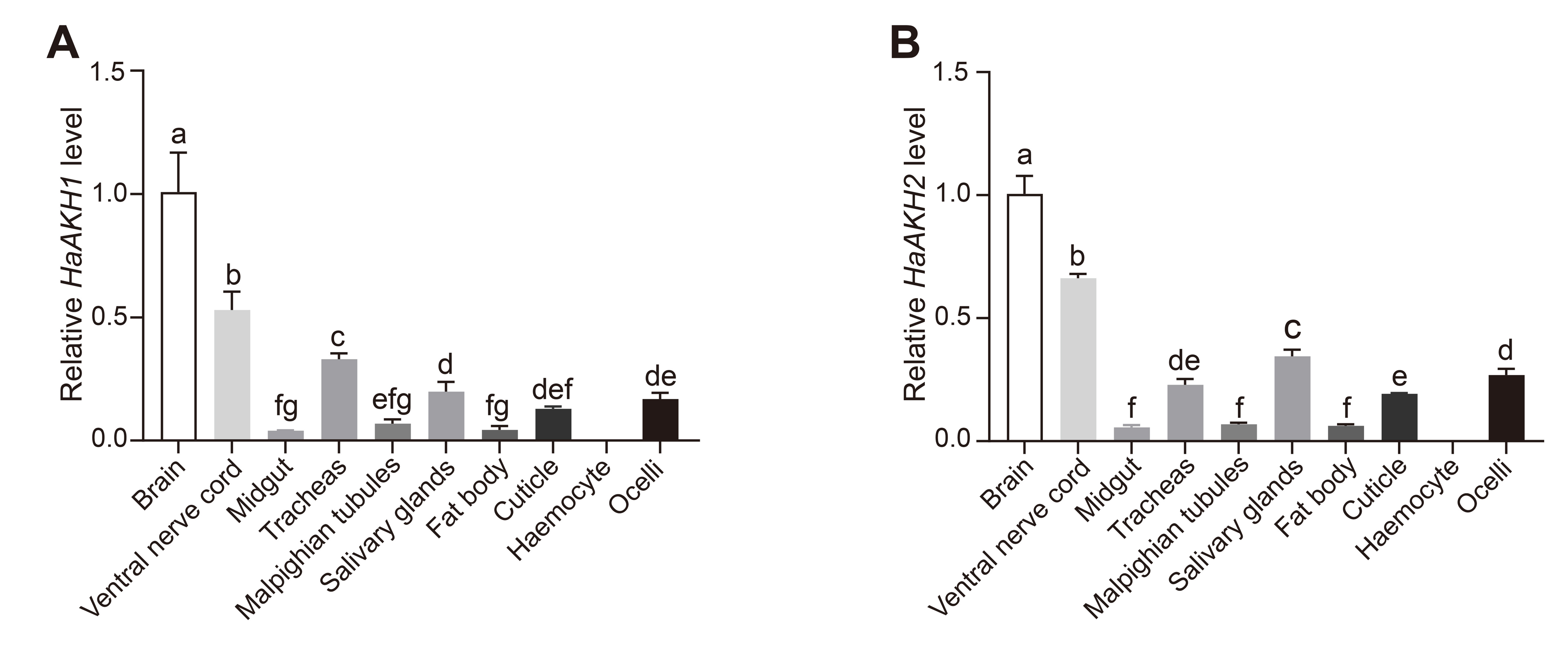

Supplement: S4 Fig — (A) Relative expression level of HaAKH1 in larvae various tissues. (B) Relative expression level of HaAKH2 in larvae various tissues. Data represented mean ± SEM. Different lowercase letters indicated significant differences among different tissues (p < 0.05). (TIF) [file ppat.1012932.s004.tif]

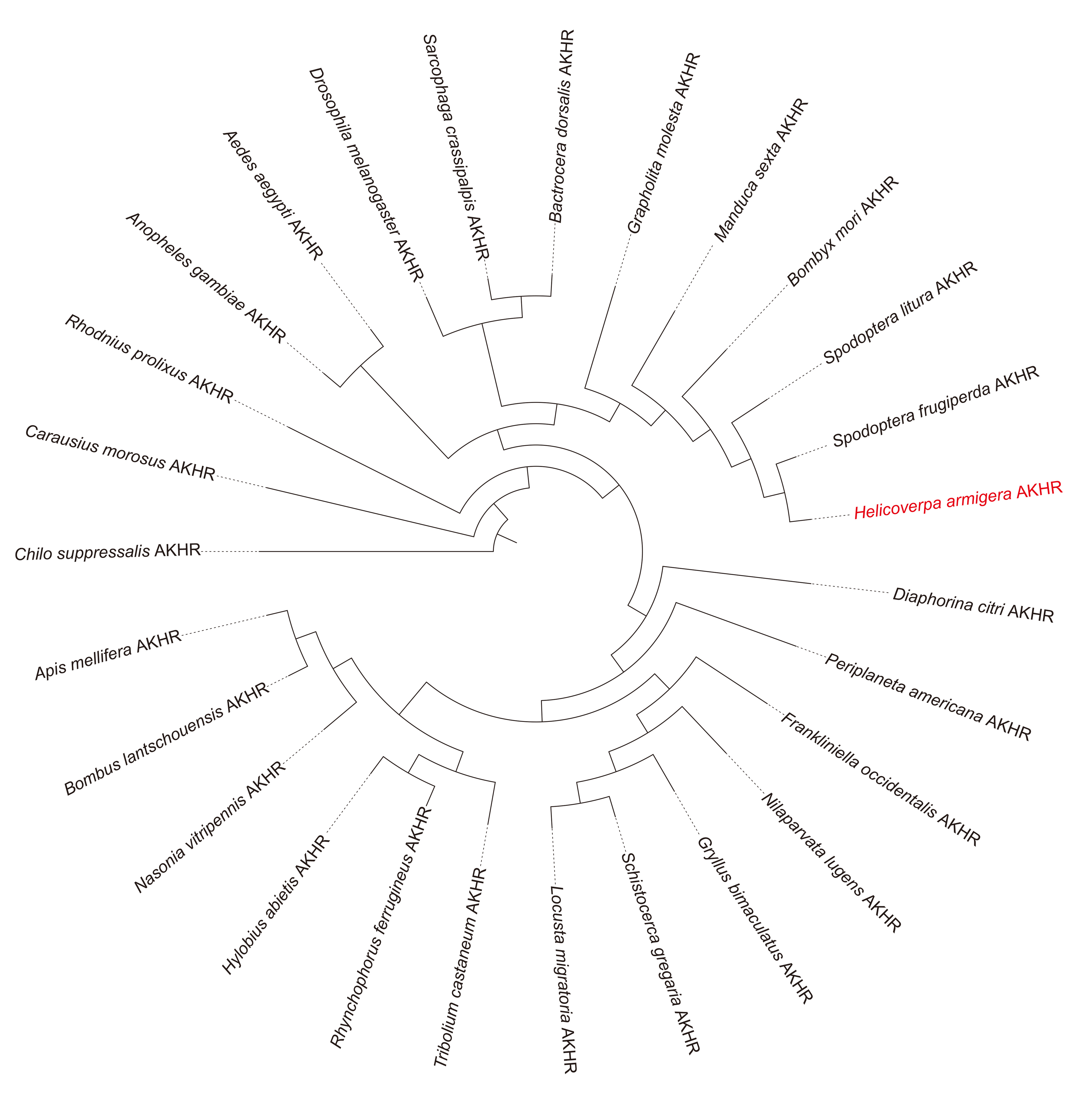

Supplement: S5 Fig — H.armigera sequences were highlighted in red. The protein names and accession numbers were listed in S3 Table. (TIF) [file ppat.1012932.s005.tif]

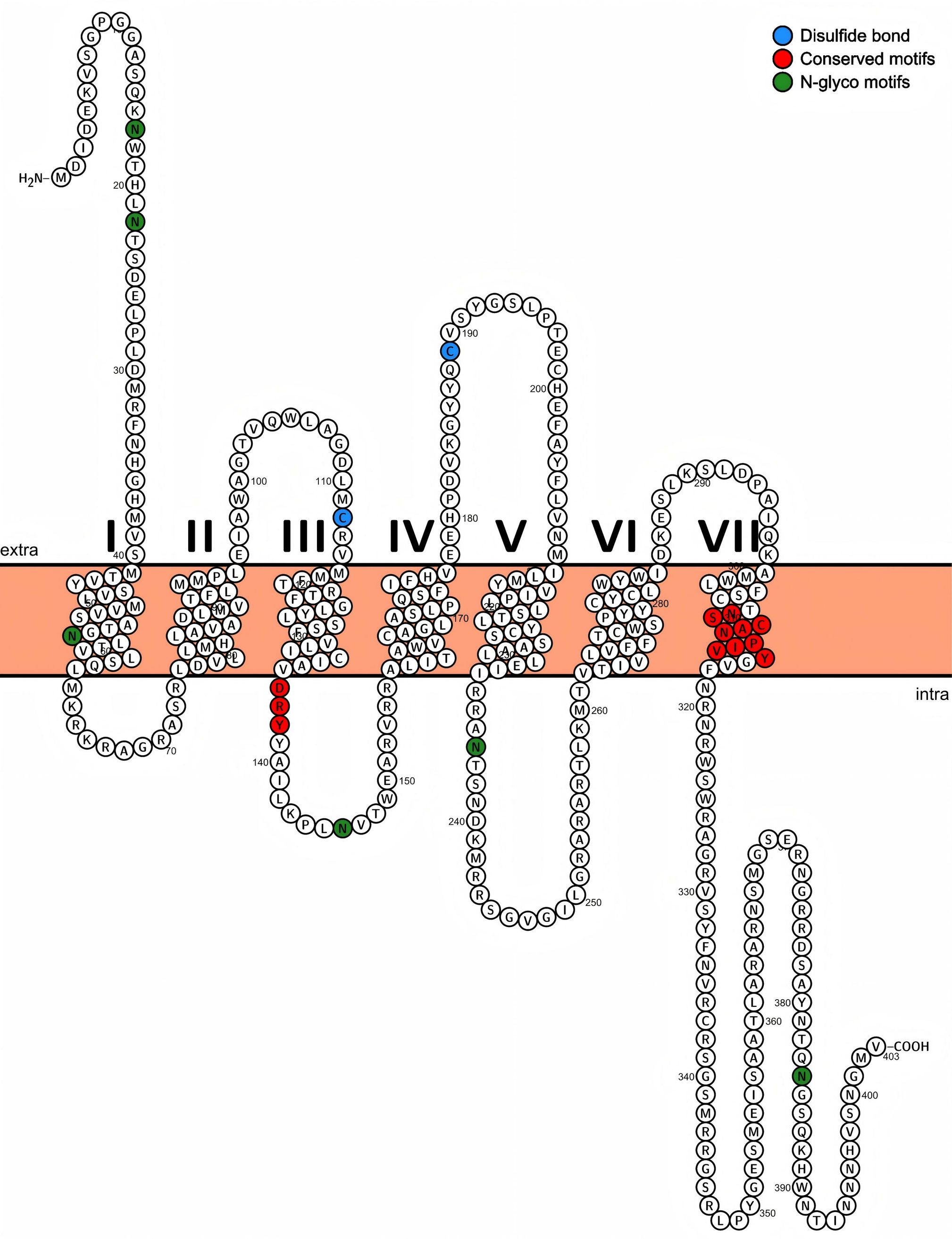

Supplement: S6 Fig — Seven transmembrane domains were located within the orange shaded cell membrane. The conserved motifs of Rhodopsin-like receptors were highlighted in red. A pair of cysteine residues that form a disulfide bond were highlighted in blue. The predicted N-glycosylation sites were highlighted in green. (TIF) [file ppat.1012932.s006.tif]

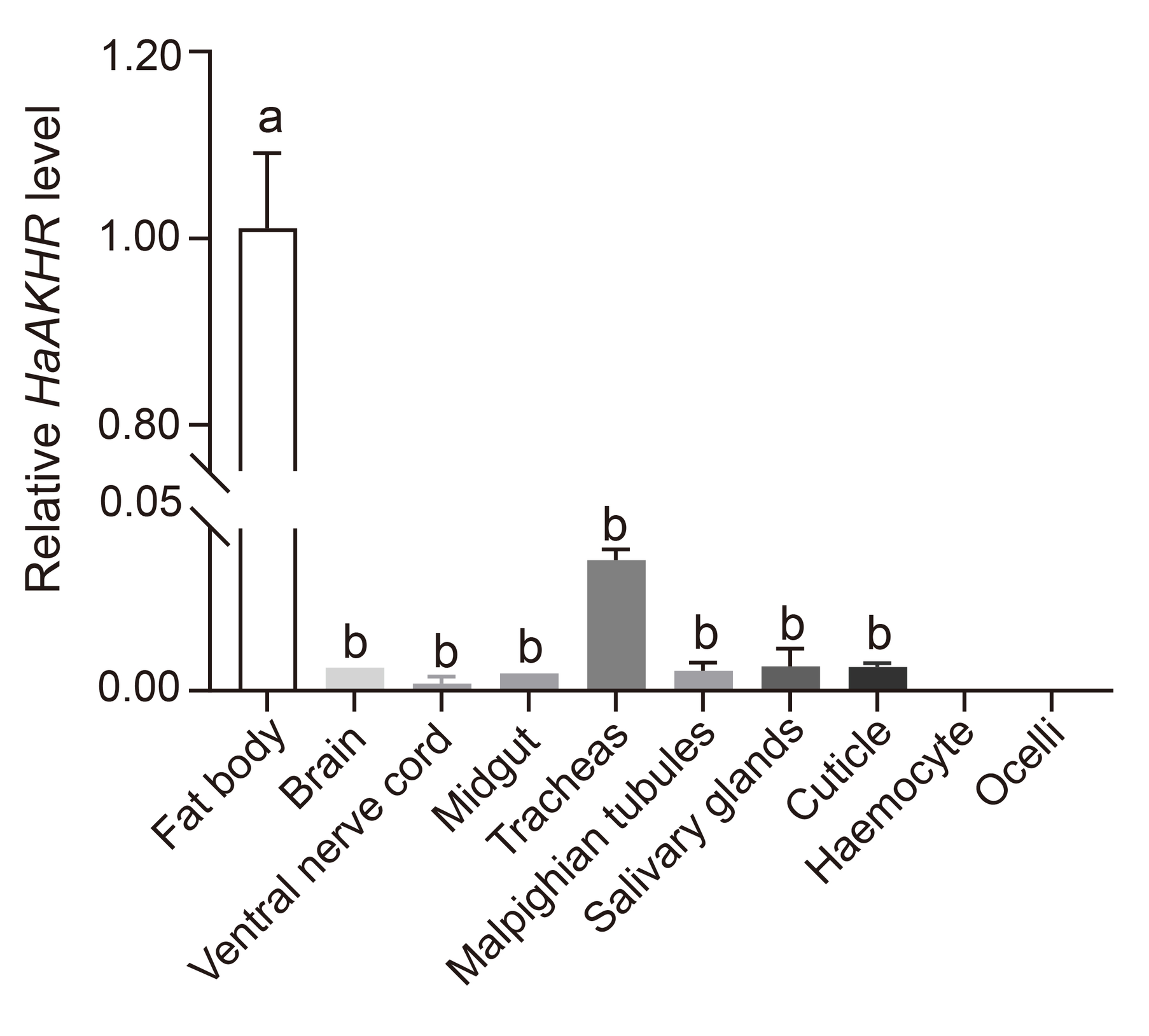

Supplement: S7 Fig — Relative expression level of HaAKHR in larvae various tissues. Data represented mean ± SEM. Different lowercase letters indicated significant differences among different tissues (p < 0.05). (TIF) [file ppat.1012932.s007.tif]
