# Supplementary material for: Baculoviruses manipulate host lipid metabolism via adipokinetic hormone signaling to induce climbing behavior
Source: PLoS Pathog. 2025 Jan 31;21(1):e1012932. doi: 10.1371/journal.ppat.1012932 (PMC11819524; doi:10.1371/journal.ppat.1012932)
Supplement: S1 Table — (DOCX) [file ppat.1012932.s008.docx]

**Table S1.** **Primers used in this study.**

| Gene | Direction | Sequence (5′-3′) | Product size (bp) |
| --- | --- | --- | --- |
| **cDNA cloning** | | |  |
| *HaAKH1* | F | ATGAAGGCCGTTAGTAT | 207 |
|  | R | TCAAGGCTTCTGGGGGCAA |  |
| *HaAKH2* | F | ATGTGTCGTATCCTGGTC | 222 |
|  | R | TTAGATTTTTCCTTCACGC |  |
| *HaAKHR* | F | ATGGACATAGACGAAAAA | 1212 |
|  | R | CTATACCATGCCATTGCT |  |
| *HaLsd1* | F | ATGGCAAAAGTTCAAAAGCC | 1128 |
|  | R | TTAGTTGAGTCCGTTAATAG |  |
| *HaLsd2* | F | ATGGCTACAGAAGTGAGC | 918 |
|  | R | TTAATTGTTAGAAGTTTC |  |
| *HaBmm* | F | ATGAACTTATCGTTTGC | 3282 |
|  | R | TCACGAGCTGTCCTCGT |  |
| *HaHSL* | F | ATGGCAATCCTACAAGTC | 3198 |
|  | R | TCATAACTGTTCGTTCTCC |  |
| **qRT-PCR** | | |  |
| *HaAKH1* | F | TTCGTCCTCGTCCTGG | 110 |
|  | R | CTCCTGCAAGACATGGTA |  |
| *HaAKH2* | F | GTGTCGTATCCTGGTCTT | 124 |
|  | R | ATCATTGGGCTGTTCG |  |
| *HaAKHR* | F | TCATACCGTTGCTGTCCAC | 91 |
|  | R | CGACGCATCTTATCATTGG |  |
| *RPL32* | F | CATCAATCGGATCGCTATG | 152 |
|  | R | CCATTGGGTAGCATGTGAC |  |
| *poly* | F | CAAACCGAACCGTTGTTACC | 171 |
|  | R | TGCAAGTTCATAACGGGAC |  |
| *HaHSL* | F | AAGGCGTTCTGGTTTCTG | 132 |
|  | R | TTTGCCCTCTATGGTCGT |  |
| *HaBmm* | F | GCAGGCAGTTGGATAG | 141 |
|  | R | GGTAGTAATAAGCGAGCA |  |
| *HaLsd1* | F | GCTCGCCTATGTTGCTGA | 208 |
|  | R | GTTCTTGGGCAAATCGTTA |  |
| *HaLsd2* | F | GGAAGAAACCAGCAATGAAA | 194 |
|  | R | GTAAAGCAGCCAGGAGGG |  |
| **RNA interference** | | |  |
| *dsHaAKH1* | F | taatacgactcactataggg CTCTGCCTGTTCGC | 176 |
|  | R | taatacgactcactataggg CGGCCTCATTCTGT |  |
| *dsHaAKH2* | F | taatacgactcactataggg CTGTTCTCATCGAGGGTC | 184 |
|  | R | taatacgactcactataggg TTTCTCCTTCCGACACG |  |
| *dsHaAKHR* | F | taatacgactcactataggg AGGGAACTTGACGGTGCT | 532 |
|  | R | taatacgactcactataggg AACGGTATGACGTACATGAGAA |  |
| *dsHaHSL* | F | taatacgactcactataggg AGTTGAACGTGCCGATACTGT | 433 |
|  | R | taatacgactcactataggg CTGATTGTTGCCGCCATT |  |
| *dsEGFP* | F | taatacgactcactatagggCAGTTCTTGTTGAATTAGATG | 476 |
|  | R | taatacgactcactatagggTTTGGTTTGTCTCCCATGATG |  |
