# Supplementary material for: Baculoviruses manipulate host lipid metabolism via adipokinetic hormone signaling to induce climbing behavior
Source: PLoS Pathog. 2025 Jan 31;21(1):e1012932. doi: 10.1371/journal.ppat.1012932 (PMC11819524; doi:10.1371/journal.ppat.1012932)
Supplement: S2 Table — (DOCX) [file ppat.1012932.s009.docx]

**Table S2.** **GenBank accession numbers used for the multiple sequence alignment and phylogenetic analysis of AKH and ACP.**

| **Insect** | **Protein** | **Acc. number** |
| --- | --- | --- |
| *Aedes aegypti* | adipokinetic hormone 1 | CAY77165.1 |
| *Agrotis ipsilon* | adipokinetic hormone 1 | C0HL91.1 |
| *Bombyx mori* | adipokinetic hormone 1 | ABW97444.1 |
| *Chilo suppressalis* | adipokinetic hormone 1 | ALM30296.1 |
| *Helicoverpa armigera* | adipokinetic hormone 1 | WGD18896.1 |
| *Papilio machaon* | adipokinetic hormone 1 | KPJ15512.1 |
| *Plutella xylostella* | adipokinetic hormone 1 | AJM76765.1 |
| *Spodoptera exigua* | adipokinetic hormone 1 | AXY04229.1 |
| *Tribolium castaneum* | adipokinetic hormone 1 | NP_001107797.1 |
| *Aedes aegypti* | adipokinetic hormone 2 | CAY77162.1 |
| *Agrotis ipsilon* | adipokinetic hormone 2 | C0HKR1.1 |
| *Bombyx mori* | adipokinetic hormone 2 | NP_001124365.1 |
| *Chilo suppressalis* | adipokinetic hormone 2 | ALM30297.1 |
| *Helicoverpa armigera* | adipokinetic hormone 2 | WGD18897.1 |
| *Papilio machaon* | adipokinetic hormone 2 | KPJ15513.1 |
| *Plutella xylostella* | adipokinetic hormone 2 | AJM76766.1 |
| *Spodoptera exigua* | adipokinetic hormone 2 | AXY04230.1 |
| *Tribolium castaneum* | adipokinetic hormone 2 | NP_001107818.1 |
| *Acyrthosiphon pisum* | adipokinetic hormone | NP_001243520.1 |
| *Anabrus simplex* | adipokinetic hormone | XP_066998441.1 |
| *Apis mellifera* | adipokinetic hormone | AEW68342.1 |
| *Diaphorina citri* | adipokinetic hormone | KAI5741393.1 |
| *Drosophila melanogaster* | adipokinetic hormone | NP_523918.1 |
| *Nilaparvata lugens* | adipokinetic hormone | BAO00932.1 |
| *Rhodnius prolixus* | adipokinetic hormone | ACZ52614.1 |
| *Aedes aegypti* | Adipokinetic hormone/corazonin-related peptide | Q16RI5.2 |
| *Bombyx mori* | Adipokinetic hormone/corazonin-related peptide | NP_001127713.1 |
| *Chilo suppressalis* | Adipokinetic hormone/corazonin-related peptide | ALM30298.1 |
| *Helicoverpa armigera* | Adipokinetic hormone/corazonin-related peptide | XP_021197899.1 |
| *Nilaparvata lugens* | Adipokinetic hormone/corazonin-related peptide | BAO00933.1 |
| *Spodoptera exigua* | Adipokinetic hormone/corazonin-related peptide | AXY04231.1 |
| *Tribolium castaneum* | Adipokinetic hormone/corazonin-related peptide | NP_001159497.2 |
