# Supplementary material for: Baculoviruses manipulate host lipid metabolism via adipokinetic hormone signaling to induce climbing behavior
Source: PLoS Pathog. 2025 Jan 31;21(1):e1012932. doi: 10.1371/journal.ppat.1012932 (PMC11819524; doi:10.1371/journal.ppat.1012932)
Supplement: S3 Table — (DOCX) [file ppat.1012932.s010.docx]

**Table S3. GenBank accession numbers used for the multiple sequence alignment and phylogenetic analysis of AKHR.**

| **Insect** | **Protein** | **Acc. number** |
| --- | --- | --- |
| *Aedes aegypti* | adipokinetic hormone receptor | CAY77164.1 |
| *Anopheles gambiae* | adipokinetic hormone receptor | ABD60146.1 |
| *Apis mellifera* | adipokinetic hormone receptor | NP_001035354.1 |
| *Bactrocera dorsalis* | adipokinetic hormone receptor | AQX83416.1 |
| *Bombus lantschouensis* | adipokinetic hormone receptor | QGN75353.1 |
| *Bombyx mori* | adipokinetic hormone receptor | NP_001037049.1 |
| *Carausius morosus* | adipokinetic hormone receptor | QRN45460.1 |
| *Chilo suppressalis* | adipokinetic hormone receptor | ALM88324.1 |
| *Diaphorina citri* | adipokinetic hormone receptor | WKC57600.1 |
| *Drosophila melanogaster* | adipokinetic hormone receptor | NP_477387.1 |
| *Frankliniella occidentalis* | adipokinetic hormone receptor | KAE8753142.1 |
| *Grapholita molesta* | adipokinetic hormone receptor | QPZ46758.1 |
| *Gryllus bimaculatus* | adipokinetic hormone receptor | ADZ17179.1 |
| *Helicoverpa armigera* | adipokinetic hormone receptor | XP_021200810.1 |
| *Hylobius abietis* | adipokinetic hormone receptor | AVI00624.1 |
| *Locusta migratoria* | adipokinetic hormone receptor | ANW09575.1 |
| *Manduca sexta* | adipokinetic hormone receptor | ACE00761.1 |
| *Nasonia vitripennis* | adipokinetic hormone receptor | NP_001161243.1 |
| *Nilaparvata lugens* | adipokinetic hormone receptor | AZP54622.1 |
| *Periplaneta americana* | adipokinetic hormone receptor | AAQ17230.1 |
| *Rhodnius prolixus* | adipokinetic hormone receptor | AIJ49751.1 |
| *Rhynchophorus ferrugineus* | adipokinetic hormone receptor | QGA72493.1 |
| *Sarcophaga crassipalpis* | adipokinetic hormone receptor | AOC38019.1 |
| *Schistocerca gregaria* | adipokinetic hormone receptor | AVG47955.1 |
| *Spodoptera frugiperda* | adipokinetic hormone receptor | UZC46996.1 |
| *Spodoptera litura* | adipokinetic hormone receptor | UPG19308.1 |
| *Tribolium castaneum* | adipokinetic hormone receptor | NP_001076809.1 |
