# Supplementary material for: Baculoviruses manipulate host lipid metabolism via adipokinetic hormone signaling to induce climbing behavior
Source: PLoS Pathog. 2025 Jan 31;21(1):e1012932. doi: 10.1371/journal.ppat.1012932 (PMC11819524; doi:10.1371/journal.ppat.1012932)
Supplement: Attachment — Submitted filename: Response to Reviewers.docx [file ppat.1012932.s011.docx]

Dear editors and reviewers,

Thank you for your decision letter and comments concerning our manuscript entitled “Baculoviruses manipulate host lipid metabolism via adipokinetic hormone signaling to induce climbing behavior” (Manuscript ID PPATHOGENS-D-24-02518). We would like also to thank you for allowing us to resubmit a revised copy of the manuscript. All comments were valuable and have considerably helped us in revising and improving our manuscript and clarifying the significance of our study. Based on the instructions provided in the decision letter and comments provided by the reviewers, we have carefully analyzed all the comments and have accordingly made revisions, which we hope meet your approval. Also appended to this letter are point-by-point responses to the comments raised by the reviewers.

Part I - Summary

Please use this section to discuss strengths/weaknesses of study, novelty/significance, general execution and scholarship.

Reviewer #1: This is a detailed and very thorough analysis of the role of lipid metabolism in baculovirus-induced climbing behavious of infected larvae. The results provide new and interesting data to help explain why baculovirus induces climbing behaviour by manipulating energy sources, although the link to why climbing is impacted rather than just crawling remains elusive. Overall, this is of significance not just to those that study baculoviruses but more broadly as well.

**Response:** Many thanks for your positive words and your comments that helped to improve our manuscript.

Overall the study appears to have been carefully carried out with appropriate data and controls. However, the nature of the figures with many sub-panels means that the imaging data is quite hard to discern in the very small panels. Much is made of the difference in size observed for lipid droplets but sometimes this is hard to distinguish. The use of the pink and orange for colours is mostly suitable but in some line graphs e.g. Fig 3A, it is hard to see the difference between the orange and the pink. I wonder if one of the colours should be changed to something more distinctive?

**Response:** Thank you for your excellent suggestions on graphs. According to your comments, we have made the following revisions:

1. We have increased the magnification and clarity of the lipid droplet images, and conducted statistical and differential analysis on the size of the lipid droplets (Figure 1, 2, 5 and 6).

2. We have replaced orange with green to better distinguish it from pink (Figure 2, 3, 4 and 6).

The abstract is clear and concise except for the phrase in line 39/40 - this is ambiguous and as written could mean the height of the larvae rather than the height travelled or reached by the larvae.

**Response:** We have revised according to the suggestions (Line 39/40). And what we want to express is that the final death height of the larvae has decreased.

The materials and methods section is clear and sufficiently detailed.

The results section is well presented - I picked up a few typogaphical errors, minor mistakes in English grammar that should be corrected e.g. 277 suggesting enhanced (the not needed); 304 post-infection; 303 & 305, 'in' infected larvae (not 'of'); 306, remove and after the comma; 332 (Figure S4 'and' Table S3).

**Response:** Thanks for your careful review. We have revised according to the suggestions (L295, L314, L313&315, L316, L342).

I liked the use of the schematic to put forward the proposed mechamism of action.

279 - Why was coconut oil used to increase lipds in the diet?

**Response:** We referred to the methods for making high-fat diet in the previous studies. Coconut oil can be absorbed and utilized by insects, and has a low melting point that can be melted by heating. When making diet, coconut oil can be mixed evenly with other substances, and after cooling, it forms a solid form together with other substances, which is easy to feed. However, other plant oils have a high melting point and do not form a solid at an insect rearing temperature of 26 ℃, making it difficult to mix evenly with diet.

Birse RT, Choi J, Reardon K, Rodriguez J, Graham S, Diop S, Ocorr K, Bodmer R, Oldham S. High-fat-diet-induced obesity and heart dysfunction are regulated by the TOR pathway in *Drosophila*. Cell Metab. 2010;12(5):533-44.

Huang R, Song T, Su H, Lai Z, Qin W, Tian Y, Dong X, Wang L. High-fat diet enhances starvation-induced hyperactivity via sensitizing hunger-sensing neurons in *Drosophila*. eLife. 2020; 9: e53103.

319/320 - Reference for enhancement of crawl

**Response:** Here, 'HearNPV infection can enhance the crawling distance of larvae' is a description based on our experimental results, and the specific results can be seen in Figure 3C. Previous studies have reported that baculoviruses can induce ‘hyperactivity’ in infected hosts.

Kamita, SG, Nagasaka, K, Chua, JW, Shimada, T, Mita, K, Kobayashi, M, Maeda, S, Hammock, BD. A baculovirus-encoded protein tyrosine phosphatase gene induces enhanced locomotory activity in a Lepidopteran host. Proc Natl Acad Sci U S A. 2005;102: 2584-2589.

van Houte, S, Ros, VID, Mastenbroek, TG, Vendrig, NJ, Hoover, K, Spitzen, J, van Oers, MM. Protein tyrosine phosphatase-induced hyperactivity is a conserved strategy of a subset of baculoviruses to manipulate Lepidopteran host behavior. PLoS One. 2012; 7: e46933.

Reviewer #2: In this study, the authors investigate how baculovirus influences lipid metabolism in infected larvae and the subsequent behavioral changes, particularly climbing behavior, which is believed to facilitate virus transmission. Using various techniques, the authors have demonstrated that manipulating lipid metabolism through HaAKH signaling not only impacts the survival and replication of the virus but also enhances the climbing behavior of the larvae. They have also shown that hormone-sensitive lipase activity is regulated by HaAKH signaling and is essential for the climbing behavior induced by the virus. The findings of this study offer insights into the molecular mechanisms by which baculoviruses manipulate host behavior through lipid metabolism. The authors propose a model in which baculoviruses hijack AKH signaling to enhance lipid metabolism, thereby promoting their own replication and inducing climbing behavior in infected larvae. This research expands the understanding of host-parasite interactions. Although the topic investigated is intriguing, there are several issues with writing, describing methodology procedures, and presenting results that require serious revision (possibly necessitating a revision and resubmission).

**Response:** We appreciated your comments which have helped us to further improve the manuscript and we have carefully analyzed all the comments and have accordingly made revisions.

Reviewer #3: Climbing behavior is a renowned phenotype for caterpillars when they were infected by baculoviruses. However, the underlying molecular mechanisms are largely unclear. Zhu et al., performed the experiments to investigate how Helicoverpa armigera single nucleopolyhedrovirus (HearNPV) influences host behavior, specifically promoting climbing behavior in infected larvae through manipulation of lipid metabolism. They further linked host lipid metabolism to behavioral changes, focusing on the roles of two adipokinetic hormones (HaAKH1 and HaAKH2) and their receptor, HaAKHR. One of the key findings is that hormone-sensitive lipase (HaHSL) acts as a downstream effector of HaAKH signaling, bridging the gap between AKH receptor and changes of host lipid droplets. Their findings provided novel insights into how HearNPV affects its host's climbing behavior. Overall, I really enjoyed reading this manuscript and thought this study is interesting and well-done.

**Response:** Thanks a lot for your positive comments and valuable suggestions on our manuscript, and we have carefully considered and revised the issues pointed out.

Part II – Major Issues: Key Experiments Required for Acceptance

Please use this section to detail the key new experiments or modifications of existing experiments that should be absolutely required to validate study conclusions.

Reviewer #1: I have no major issues with this paper.

Reviewer #2: L271: How were softness and shallowness measured? These aspects are not evident in the figure. The figure should be informative, and the differences should be highlighted using arrowheads, etc.

**Response:** Thank you for pointing out this issue. We initially showed the changes in the bodies of infected larvae in order to illustrate that the bodies became soft and swollen after infection, possibly due to changes in fat bodies, which are the main fillers in insect bodies. During our long-term experiments, it was evident that the bodies of infected larvae became softer. Unfortunately, we attempted to quantify the softness of the larvae' bodies using various methods, but ultimately did not find a suitable one. Therefore, we chose more intuitive photos for display. As shown in Figure A, after infection, the bodies of infected larvae can be flattened and bent to a greater extent, indicating that the body of insects become softer. To further demonstrate the changes in fat bodies, we dissected and photographed the fat bodies of healthy and infected larvae. As shown in Figure B, the fat bodies of infected larvae became more fragmented, which intuitively indicates that the virus caused changes in the lipid metabolism of host larvae. Taking into account your suggestions and the relevance of the results, we have included this part of the results in Figure S1 (L289-290).

Figures 1, 2, 5, and 6: The indicated differences in the microscopy figures are unclear. The pointed differences should be clearly indicated. Perhaps microscopic images with higher magnifications would better facilitate the comparison of differences.

**Response:** Thanks for your valuable suggestion. We have improved the clarity of the microscopy figures in Figures 1, 2, 5, and 6. In addition, we have conducted quantitative comparison of the size of the lipid droplets to better evaluate the differences in Figures 1, 2, 5, and 6, and the detailed methods have been added to the Materials and Methods section (L126-127).

Reviewer #3: 1. In Figures 1, 2, 5, and 6, the authors compared the size of larval lipid droplets, but only showed some representative images. A more thorough quantitative comparison is necessary to strengthen the conclusions and the n value should be provided. Specifically, it would be helpful to include measurements of the lipid droplet size to better evaluate the observed changes, please also provide the detailed methods in the Method section.

**Response:** Thanks for your valuable suggestion. We have measured the area of at least 200 lipid droplets for each treatment to conduct quantitative comparison of the size of the lipid droplets to better evaluate the differences in Figures 1, 2, 5, and 6, and the detailed methods have been added to the Materials and Methods section (L126-127).

2. The authors found that the crawling distance of dsHaHSL-treated healthy larvae was decreased compared with the dsEGFP-treated healthy larvae. How about the healthy larvae that dsHaAKH1-treated, dsHaAKH2-treated or dsHaAKHR-treated? It needs to be clear whether dsHaAKH or dsHaAKHR alone can cause changes in crawling ability.

**Response:** Thanks for your suggestions. We have conduted RNAi the *HaAKH1*, *HaAKH2*, and *HaAKHR* of healthy larvae and found that the crawling distance of the insects decreased after RNAi, consistent with the results of infected larvae. In addition, to further confirm this conclusion, muture peptides HaAKH1 and HaAKH2 were also injected into healthy larvae, resulting in an increase in crawling distance. These results have been added to the graph (Figure 3, 5 and 6).

Part III – Minor Issues: Editorial and Data Presentation Modifications

Please use this section for editorial suggestions as well as relatively minor modifications of existing data that would enhance clarity.

Reviewer #1: Minor issues and data presentation suggestions have been incorporated into the main review section.

**Response:** We have also responded and made carefully revisions to the suggestions raised by the reviewer in the main review section.

Reviewer #2: Some parts of the introduction appear fragmented and require revision for better cohesion.

**Response:** Thank you for your careful review. We have carefully revised the introduction as according to your valuable suggestions.

L70: triacylglycerol (TAG)

**Response:** revised (L78).

L87: interactions between parasites and hosts; for instance, injecting AKH into locusts infected with Metarhizium anisopliae accelerated the ...

**Response:** revised (L87-89).

L108: established from what? A natural population or a lab-reared one?

**Response:** The colony of *H. armigera* was established from a natural population in Zhengzhou, Henan Province. We have revised in the supplemented it in the manuscript (L104).

L110: larvae were reared ...

**Response:** revised (L107).

L112: moved or transferred?

**Response:** We have changed 'removed' to 'transferred' (L108).

L118: How were the larvae fed? Force-feeding or feeding via an artificial diet?

**Response:** We use a pipette to add 2 μl of the virus suspension next to the mouthparts of the larvae, and the larvae will actively feed on the droplets in a short period of time.

L123-129: Is it feasible to conduct standing and microscopy observations without preparing thin sections of the tissue (microtome)?

**Response:** Yes, it is. Firstly, due to the large size of *H.armigera* larvae, it is relatively easy to dissect the fat body separately. The fat body is white and the dissected fat body dispersed into thin flakes in PBS solution, making it easy to directly stain. In addition, we added Triton X-100 to promote dye entry into tissues. The stained fat body can be directly spread on the glass slide, and the fat body is evenly distributed on the slide without obvious overlap, which does not affect subsequent observation. And we also referred to other studies that have stained insect lipid droplets. Most insects that are easy to dissect use directly dissecting the fat body and then conduct standing and microscopy observations without preparing thin sections of the tissue. Based on literature review and our practical experience, it is feasible to conduct standing and microscopy observations without preparing thin sections of the tissue.

Du B, Ding D, Ma C, Guo W, Kang L. Locust density shapes energy metabolism and oxidative stress resulting in divergence of flight traits. Proc Natl Acad Sci U S A. 2022;119:e2115753118.

Li J, Holford P, Beattie GAC, Wu S, He J, Tan S, et al. Adipokinetic hormone signaling mediates the enhanced fecundity of *Diaphorina citri* infected by ‘*Candidatus* Liberibacter asiaticus’. eLife. 2024;13:RP93450.

L130: free fatty acids (FFA)

**Response:** revised (L128).

L134-137: This section needs to be rewritten. It appears that the kit protocol has been copied without any modifications!

**Response:** According to this comment, we have rewritten this section to include detailed experimental methods (L132-147).

L145: the height climbed by the larvae was recorded...

**Response:** revised (L155).

L149: infected H. armigera larvae instead of treated H. armigera larvae

**Response:** Because our experiment included crawling assays of healthy *H. armigera*, we feel that ‘treated’ may be more appropriate here.

L176: qPCR instead of qRT-PCR; as cDNA is synthesized beforehand.

**Response:** Thanks for pointing it out and we have revised according to the suggestions (L186).

L180: RT-qPCR instead of qRT-PCR; as cDNA synthesis and qPCR were performed separately. Please correct this throughout the manuscript.

**Response:** Thanks for pointing it out and we have revised throughout the manuscript according to the suggestions (L181, 190, 215, 230).

L188: poly?

**Response:** We have revised as “polyhedrin (poly) protein” (L198).

L196: What are the sizes of the dsRNAs? How were they diluted? Please provide more details.

**Response:** Thank you for your suggestion. We have supplemented the synthesis method of dsRNA in the manuscript (L206) and the Supplementary materials Table S1. The size of dsRNA is as follows: ds*HaAKH1* (176bp), ds*HaAKH2* (184bp), ds*HaAKHR* (532bp), ds*HaHSL* (433bp), ds*EGFP* (476bp), and we have added this part to the Supplementary materials Table S1. All dsRNAs were diluted with DEPC water (L210).

L216: ... and 2 µl was injected into the...

**Response:** revised (L227).

L235: Why did the authors use HEK293T cells instead of insect-specific cells like HZFB cells?

**Response:** HEK293T cells can be used for heterologous expression of ion channels and cell membrane receptors, typically for studying G protein coupled receptor (GPCR) signaling pathways. The HaAKHR in our study is a GPCR, so it is appropriate to use HEK293T cells for research. We have also tried using insect-specific cell S2 for experiments, but the transfection effect was not ideal. In addition, insect cells are mostly in a suspended state, and adding ligands during imaging may cause cell movement, resulting in poor imaging results. However, HEK293 cells are relatively adherent, and the transfection effect is also relatively good. Therefore, we ultimately chose HEK293 cells for the experiment.

Zhu C, Huang H, Hua R, Li G, Yang D, Luo J, Zhang C, Shi L, Benovic JL, Zhou N. Molecular and functional characterization of adipokinetic hormone receptor and its peptide ligands in *Bombyx mori*. FEBS Lett. 2009;583(9):1463-8.

L252-258: This section needs to be rewritten. It seems that the kit protocol has been copied without any modifications!

**Response:** According to this comment, we have rewritten this section to include detailed experimental methods (L63-277).

L271: How were softness and shallowness measured? These aspects are not evident in the figure. The figure should be informative, and the differences should be highlighted using arrowheads, etc.

**Response:** Thank you for pointing out this issue. We initially showed the changes in the bodies of infected larvae in order to illustrate that the bodies became soft and swollen after infection, possibly due to changes in fat bodies, which are the main fillers in insect bodies. During our long-term experiments, it was evident that the bodies of infected larvae became softer. Unfortunately, we attempted to quantify the softness of the larvae' bodies using various methods, but ultimately did not find a suitable one. Therefore, we chose more intuitive photos for display. As shown in Figure A, after infection, the bodies of infected larvae can be flattened and bent to a greater extent, indicating that the body of insects become softer. To further demonstrate the changes in fat bodies, we dissected and photographed the fat bodies of healthy and infected larvae. As shown in Figure B, the fat bodies of infected larvae became more fragmented, which intuitively indicates that the virus caused changes in the lipid metabolism of host larvae. Taking into account your suggestions and the relevance of the results, we have included this part of the results in Figure S1 (L289-290).

Figures 1, 2, 5, and 6: The indicated differences in the microscopy figures are unclear. The pointed differences should be clearly indicated. Perhaps microscopic images with higher magnifications would better facilitate the comparison of differences.

**Response:** Thanks for your valuable suggestion. We have improved the clarity of the microscopy figures in Figures 1, 2, 5, and 6. In addition, we have conducted quantitative comparison of the size of the lipid droplets to better evaluate the differences in Figures 1, 2, 5, and 6, and the detailed methods have been added to the Materials and Methods section (L126-127).

L289-299: This section is unnecessary and can be moved to supplementary texts.

**Response:** Thanks for your suggestion. We have streamlined this part of the content (L307-310).

L304: Following the successful knockdown of HaAKH1 and HaAKH2 in the infected larvae...

**Response:** revised (L314-315).

L316: RNAi of these genes and the injection of their mature peptide into the larvae.

**Response:** revised (L326).

L322: ...in the height climbed before death.

**Response:** revised (L332).

L326-327: This seems contradictory! These parameters should be increased, as shown in the figures.

**Response:** Thank you for pointing it out, this is our writing error. We have corrected the ‘decreased’ to ‘increased’ (L337).

L328-348: The interaction of HaAKH with HaAKHR is evident. Did the authors anticipate a different result?

**Response:** Our study is the first to report the presence of two *AKH* genes, *HaAKH1* and *HaAKH2*, in *H. armigera*, and the identification of *AKH* and *AKHR* was predicted based on reports from other insects. To confirm that the predicted *AKH* and *AKHR* are indeed the *AKH* signaling system of *H. armigera*, we validated the activation of AKHR by AKH1 and AKH2 using molecular docking and Ca^2+^ imaging experiments. This results strongly prove the reliability of the HaAKH signal system in our research.

L349: “HaAKHR regulated lipid metabolism” instead of the current title.

**Response:** revised (L359).

L418: It is unclear whether Candidatus Liberibacter asiaticus can be considered a parasite. It is a plant pathogen vectored by Diaphorina citri.

**Response:** Thanks for pointing it out, and we have already deleted this section.

L426-436: This section is not particularly relevant to the discussion.

**Response:** We have rewritten this section. We would like to emphasize that we have validated for the first time the existence of the AKH signaling system (consist of HaAKH1, HaAKH2 and HaAKHR) and the interaction of HaAKH1, HaAKH2 with HaAKHR in *H.armigera*. (L431-443)

L437: This should be deleted.

**Response:** revised (L444).

Reviewer #3: 1. The authors report interference efficiency at 24 and 48 hours after injection, with two time points showing around 50% efficiency, indicating effective interference within 48 hours. However, in the Methods section (lines 208-211), the second injection is 48 hours after the first shot. Is this a mistake? In addition, the authors should clarify why this specific time point was chosen for the second injection.

**Response:** The second injection was indeed 48 hours after the first injection. In our experiment, the observation of climbing behavior lasted for a relatively long time, in order to ensure the sustained function of dsRNAs, we chose to perform a second injection. For climbing behavior assay, we put the injected larva into a closed glass tube to observe the climbing behavior. Therefore, it is more appropriate to perform a second injection before placing the larvae into the tubes. In addition, 2.5 days post the first injection, the death of the infected larvae began to occur. Therefore, we need to put the larvae into the climbing tubes no later than 2 days post the first injection, which is 48 hours post the first injection. Taking into account the above considerations, we conducted a second injection at 48 hours post the first injection.

2. In the Crawling assays, there is a discrepancy between the CK-dsEGFP group in Figure 3C and the CK+dsEGFP group in Figure 6L, both representing healthy larvae treated with dsEGFP. This difference is substantial and affects the interpretation of the study’s conclusions. The authors should address the reasons behind this discrepancy between the two controls.

**Response:** Thank you for pointing out this issue. To address this issue, we repeated all crawling experiments and measured the crawling distance for at least 50 larvae for each treatment. The specific results have been added to the Figures (Figure 3, Figure 5 and Figure 6). Through repeated experiments, we found no problem with our previous conclusion that HearNPV infection promotes larval crawling, and *HaAKH1*, *HaAKH2*, *HaAKHR*, and *HaHSL* can all play a role in promoting crawling. The difference between the two CK-EGFP results presented may be due to different batches of insects. But both the control and treatment groups for the same experiment ensured the use of the same batch of insects, so although there were differences in specific crawling distance, the final results were reliable.

3. There is inconsistency in the use of "hpi" and "dpi" across different figures, with some figure legends also reversing these terms. It is recommended to standardize the terminology and use "dpi" consistently throughout the manuscript.

**Response:** Thanks for your careful review. We have standardized the format to “dpi” throughout the manuscript.

4. In Figure 1E-G and Figure 6L, the notation "xx+xx" is used in the figures, while "xx-xx" is used in the figure legends. It is necessary to use the same format.

**Response:** Thanks for your careful review. We have standardized the format to “xx+xx”.

5. Figure S1 does not indicate statistical significance, which should be addressed.

**Response:** Thanks for pointing it out, and we have indicated statistical significance in Figure S2.

6. The image of western blot does not indicate the hours post injection.

**Response:** The western blot was conducted at 48 hours post injection and we have indicated it in the legends of Figure 3 and Figure 5.

7. Line 379-381 describe the results of Figure 6L, not Figure 6K. There is no description of Figure 6K in Results.

**Response:** Thanks for pointing it out, and we have corrected this mistake and added a description of Figure 6K (L388).

8. In Line 415, the reference format differs from the rest of the manuscript. Please ensure that all references are consistently formatted.

**Response:** Thanks for pointing it out, and we have corrected this mistake (L425).
